# Commentary: Conservation of *AtTZF1, AtTZF2*, and *AtTZF3* homolog gene regulation by salt stress in evolutionarily distant plant species

**DOI:** 10.3389/fpls.2016.00254

**Published:** 2016-02-24

**Authors:** Bhaskar Gupta, Atreyee Sengupta, Kamala Gupta

**Affiliations:** Plant Biotechnology Laboratory, Department of Biological Sciences, Presidency University, KolkataKolkata, India

**Keywords:** CCCH zinc finger proteins, abiotic stress tolerance, arginine-rich tandem zinc-finger proteins, salinity stress tolerance, durum wheat

In the research article entitled “Conservation of *AtTZF1, AtTZF2*, and *AtTZF3* homolog gene regulation by salt stress in evolutionary distant plant species”, D'Orso et al. (2015) have presented the genomic characterization of two homeologous zinc finger genes called *TdTZF1-A* and *TdTZF1-B*. The authors have utilized various bioinformatic and molecular tools to establish that *Arabidopsis* zinc finger genes *AtTZF1-2-3* are putative orthologs of the durum wheat *TdTZF1-A* and *TdTZF1-B* genes. Additionally they have shown that zinc finger protein AtTZF3 is a negative factor controlling *Arabidopsis* seed germination in the presence of salt. Moreover, they have indicated the presence of highly conserved AtTZF1-2-3-like proteins in phylogenetically distant species such as bryophytes. The authors have thus argued that the function of AtTZF1-2-3-like proteins in regulating seed germination emerged from their role in pre-existing NaCl-stress signaling pathways controlling growth and development in lower plants. Expression level analysis of these genes during the period of seed germination and abiotic stress has revealed their functional analogy.

In *Arabidopsis*, nearly all the members of RR-TZF subfamily have been characterized and it was found that the genes coding for RR-TZF protein exhibit differential response when exposed to different abiotic stresses and other growth stimuli. Studies reveal their close interaction with ABA, GA and jasmonic acid, which is probably one of the key mechanisms behind their mode of action. Another possible mechanism adapted by RR-TZF proteins in regulating gene expression in different organisms is by binding to newly synthesized mRNAs and modulating their processing and degradation, which might play a significant role in abiotic stress adaptive responses. *In silico* analysis reveals the presence of multiple putative RNA binding residues in the RR-TZF domain which serves as the RNA binding site (Qu et al., 2014).

Zinc finger proteins comprise a large and diverse group of proteins whose members can readily form a stable finger like structure in presence of one or more zinc cations and are widely conserved across the eukaryotes. Tandem CCCH zinc finger (TZF) proteins comprise a small subfamily of zinc finger proteins that is characterized by presence of a unique tandem zinc finger domain and an arginine rich (RR) motif and hence referred as arginine rich tandem zinc finger (RR-TZF) protein (Hall, 2005). Till date only three TZF members have been characterized in humans, which is pretty low when compared with plant TZF proteins that have already been identified and characterized in much higher number. Interestingly the presence of arginine rich motif in tandem zinc finger protein is a feature unique to plants (Bogamuwa and Jang, 2014). Extensive genomic studies have confirmed their presence in *Arabidopsis thaliana, Oryza sativa, Medicago truncatula, Populas trichocarpa, Zea mays*, bryophyte *Physcomitrella patens*, algae *Chlamydomonus reinhardtii*, and more recently in durum wheat (Wang et al., 2008; Chai et al., 2012; Peng et al., 2012; Zhang et al., 2013; D'Orso et al., 2015). Expression level analyses have revealed their involvement in multiple tissue specific developmental processes as well as other regulatory activities. It was observed that in *Medicago*, poplar and maize RR-TZF genes are expressed more ubiquitously than those in *Arabidopsis*, rice and soyabean indicating their participation in more specialized functions.

D'Orso et al. (2015) have investigated conservations of genes coding for arginine rich tandem zinc finger (RR-TZF) proteins during salt stress in evolutionary distant plant species. As mentioned earlier in this commentary, functions of some RR-TZF *Arabidopsis* and rice protein as abiotic stress modulator have already been established using various wet-lab and *in silico* techniques. Major objective of this work was to gain proper insight regarding the orthologous origin and functional conservation of durum wheat RR-TZF protein, which was previously identified and have been reported as a cold responsive protein, along with other already reported RR-TZF proteins present in plant belonging to different evolutionary scale including *Arabidopsis*. Authors have initially cloned the full length genomic and cDNA sequences of durum wheat (*TdTZF1-A* and *TdTZF1-B*). Based on sequence similarity, gene structure and expression analysis, they have successfully established the orthologous origin of *Arabidopsis* and durum wheat RR-TZF proteins. They used several computational methods freely available on the internet to identify the RR-TZF proteins present in the genome of 54 different plant species ranging from green algae to angiosperm using the BLASTP program with protein sequence of *Arabidopsis* tandem zinc finger proteins as the query sequence. They also constructed a phylogenetic tree with 9 different plant species belonging to different evolutionary scale in order to reveal the evolutionary relationship among the RR-TZF proteins. Along with this *in silico* analysis, they also deduced the expression level analysis of RR-TZF gene in durum wheat, *Arabidopsis* and *P. patens*, when exposed to different abiotic stresses during different developmental stages, and also constructed an overexpressing and attenuated *Arabidopsis* line for *AtTZF3* gene. To our knowledge this is the first study of this kind dealing with comprehensive analysis of RR-TZF proteins belonging to different plant genera. It makes a significant contribution in understanding the evolutionary conservation of this group of genes during the evolutionary development of both non seed and seed bearing plants and how their functions are conserved and maintained through structural integrity of conserved motifs irrespective of its phylogenetic status.

However, the lacunae lie in the fact that their mode of action is still not completely elucidated. That they play an important role in regulating seed germination and abiotic stress response is clear from all the above evidences, but exactly “how” is yet to be determined. The future work should focus on decoding this particular “how” which will enhance our knowledge and enable us to draw a complete picture.

Various studies with mammalian TZF proteins reveal their close association with cytoplasmic mRNP complexes known as processing-bodies and stress granules, which contribute essentially in post transcriptional and epigenetic regulation of gene expression by triggering mRNA degradation and post translational repression by binding with them (Anderson and Kedersha, 2009). Presence of TZF protein associated processing bodies and stress granules have also been detected in both arabidopsis and rice cells (Bogamuwa and Jang, 2013; Jan et al., 2013). They function by decapping, deadenylating, and degrading mRNA targets, thus mediating unique stress adaptive responses. D'Orso et al. (2015) have revealed that the expression of *A. thaliana*, Durum wheat and *P. patens* RR-TZF genes increase considerably with increase in environmental salt concentration, indicating their role in stress alleviation. But their primary target genes or the pathways which they regulate are yet to be discovered. Deciphering their interaction with various stress regulatory pathways such as antioxidative enzyme regulation, osmolyte biosynthesis, ion homeostasis maintenance, hormone regulations and polyamine metabolism could be an interesting avenue of further studies. Another very interesting outcome of this particular work is the role of RR-TZF encoding genes in regulating germination process both in arabidopsis and durum wheat. This is congruent with earlier studies on arabidopsis carried out by different worker (Lopez-Molina et al., 2002; Piskurewicz et al., 2008). As described earlier, D'Orso et al. (2015) have observed that these genes negatively regulate seed germination by delaying the process when exposed to salt stress. This could be an indicator of the role of RR-TZF encoding genes in developmental processes as well, thereby targeting genes that play integral role in seed germination, such as genes involved in ABA and GA signaling pathways.

Future research prospect include identification of putative target genes for wheat RR-TZF proteins, their mode of regulation and their evolution. It would also be fascinating to know whether the target genes too, like the RR-TZF proteins themselves, are evolutionarily conserved across the plant kingdom. Another very promising field of research includes detailed analysis of the crosstalk that interlinks RR-TZF proteins with both developmental and stress adaptation pathways so that the target of these proteins could be easily identified and can be used in further scientific studies (Figure 1).

**Figure 1:**
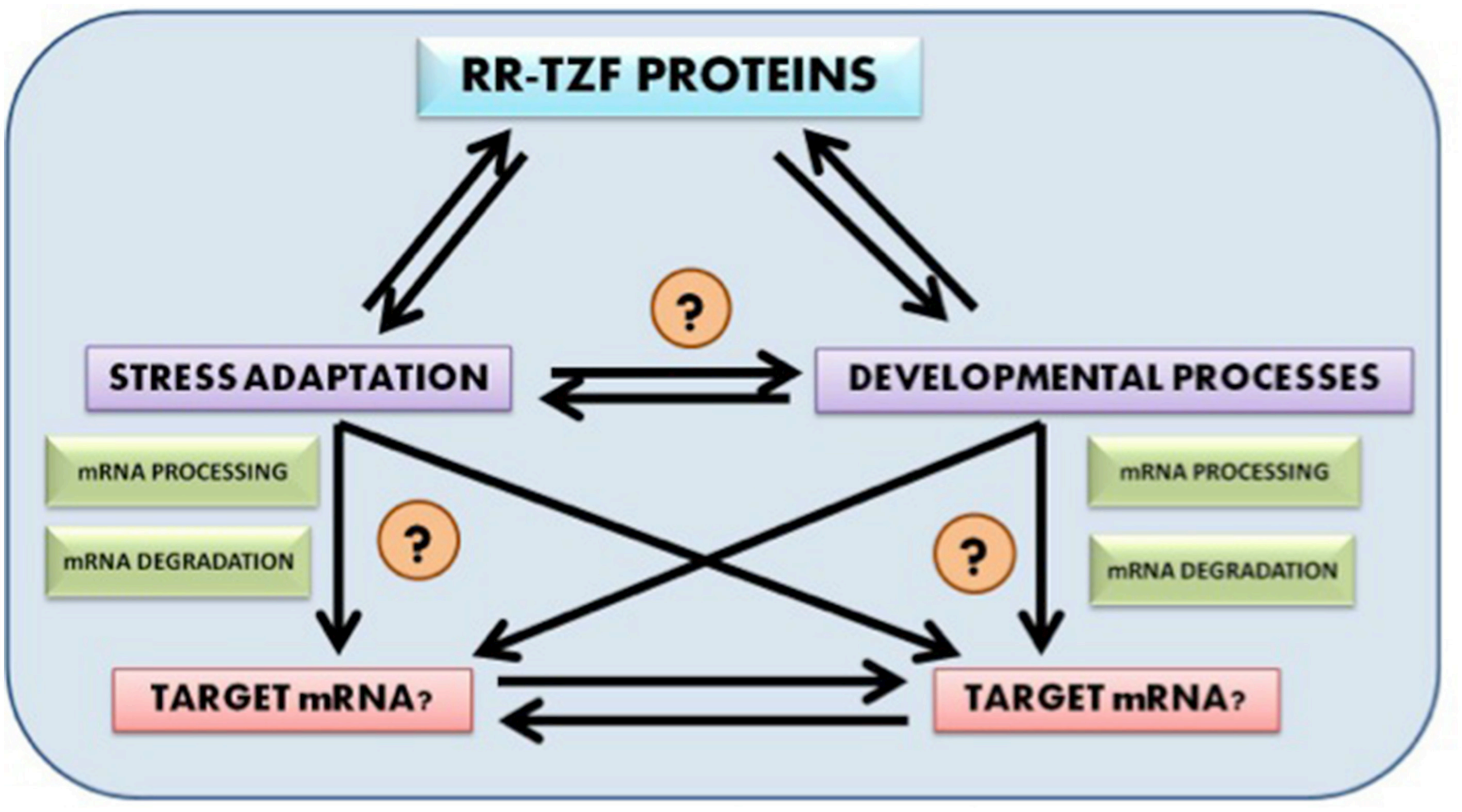
**Probable cross talk between RR-TZF proteins, stress adaptation and developmental process and their putative target identification**.

## Author contributions

All authors listed, have made substantial, direct and intellectual contribution to the work, and approved it for publication.

### Conflict of interest statement

The authors declare that the research was conducted in the absence of any commercial or financial relationships that could be construed as a potential conflict of interest.
